# Significant Acute Response of Brain-Derived Neurotrophic Factor Following a Session of Extreme Conditioning Program Is Correlated With Volume of Specific Exercise Training in Trained Men

**DOI:** 10.3389/fphys.2018.00823

**Published:** 2018-07-03

**Authors:** Emy S. Pereira, Walter Krause Neto, Atilio S. Calefi, Mariana Georgetti, Larissa Guerreiro, Cesar A. S. Zocoler, Eliane F. Gama

**Affiliations:** ^1^Laboratory of Morphoquantitative Studies and Immunohistochemistry, Department of Physical Education, São Judas Tadeu University, São Paulo, Brazil; ^2^Laboratory of Body Perception and Movement, Department of Physical Education, São Judas Tadeu University, São Paulo, Brazil; ^3^Department of Pathology, Faculty of Veterinary Medicine and Animal Science, University of São Paulo, São Paulo, Brazil; ^4^Laboratory of Human Movement, Department of Physical Education, São Judas Tadeu University, São Paulo, Brazil

**Keywords:** high-intensity interval training, effort, strength training, aerobics, cross training, neurotrophin

## Abstract

Several studies have demonstrated an acute and chronic increase of brain-derived neurotrophic factor (BDNF) in relation to different types of physical exercise. Currently, many individuals seek physical training strategies that present different types of stimulation and volume/intensity. Thus, the extreme conditioning methodology has gained great notoriety in the scientific and non-scientific environment. Knowing that BDNF values increase in an effort-dependent manner, it is necessary to study the effects of this strategy on BDNF levels. This study aimed to evaluate the acute response of BDNF in trained men submitted to an extreme conditioning program (ECP) session. Ten volunteers underwent an acute ECP session using the “as many reps as possible” (WOD-AMRAP) method, including three types of exercise (clean, wall ball and double or single-unders) for 9 min. BDNF was measured in the plasma, being collected baseline and immediately after the session. Total load of the clean exercise was five times greater than wall ball exercise (*p* < 0.05; 2096.1 ± 387.4 kg vs 415.8 ± 81.03 kg), which influenced little in the total load (*p* < 0.05, 2511.9 ± 358.52 kg) used. For the total volume, practitioners averaged 1.7 times more repetitions in the wall ball exercise compared to clean (46.2 ± 9 vs 29.5 ± 3.8 repetitions). The volunteers averaged 75.7 ± 12.6 double-unders repetitions, bringing the total volume of training to 151.4 ± 23.7 repetitions. Regarding the BDNF values, there was a significant difference (*p* = 0.05) between the pre- vs post-moments (11209.85 ± 1270.4 vs 12132.96 ± 1441.93 pg/ml). Effect size for this change as moderate (ES = 0.79). We found a positive correlation between total volume of clean exercise and delta BDNF values (*p* = 0.049). In conclusion, a single extreme conditioning session, through the practice of the WOD-AMRAP method, is capable of increasing the acute concentrations of plasma BDNF. In practical terms, we may suggest that future studies evaluate the effect of ECP as a strategy in the treatment of disorders associated with central degenerative changes.

## Introduction

Brain-derived neurotrophic factor (BDNF) is a potent neurotrophin found in many tissues, including hippocampus, skeletal muscle, cardiac muscle, and liver and adipose cells (Leibrock et al., 1989; Zafra et al., 1990; Yamamoto et al., 1996; Marosi and Mattson, 2014; Briana and Malamitsi-Puchner, 2017; Pius-Sadowska and Machaliński, 2017). In the central nervous system, this peptide stimulates neuroplasticity, through direct mediation of the formation of new neuronal circuits, neuronal survival and synaptogenesis (Gomez-Pinilla et al., 2008; Fargali et al., 2012; Numakawa et al., 2017). At the cellular level, BDNF acts via specific tyrosine kinase receptors, being directly involved in improving memory and learning in both experimental and human models (Falkenberg et al., 1992; Lapchak et al., 1993; von Bohlen Und Halbach and von Bohlen Und Halbach, 2018).

In peripheral tissues, as in skeletal muscle, BDNF appears to act in a paracrine rather than an endocrine manner (Matthews et al., 2009; Rasmussen et al., 2009; Maekawa et al., 2018). Its action, via receptor tyrosine kinase, stimulates local metabolism through enzymatic mechanisms activated by AMP-dependent kinase (Matthews et al., 2009).

Exercise and physical training seem to be potentially stimulating for the acute and chronic response of BDNF (Ferris et al., 2007; Seifert et al., 2010; Yarrow et al., 2010; Marquez et al., 2015; Liu and Nusslock, 2018). The majority of research investigating BDNF response to physical activity and exercise/physical training primarily examined aerobic exercise (Mackay et al., 2017; Liu and Nusslock, 2018). Acute increases in plasma BDNF seems to be related to effort duration and intensity (Ferris et al., 2007; Rojas et al., 2010; Seifert et al., 2010; Cho et al., 2012; Marquez et al., 2015). Literature data suggest that the higher volume, or its association with higher intensities, may influence the acute response of BDNF to aerobic exercise (Ferris et al., 2007; Cho et al., 2012).

Recent data suggest that resistance and/or concurrent training may also potentially stimulate plasma levels of neurotrophins (Cho et al., 2014; Church et al., 2016). However, an increasing number of individuals are seeking a type of exercise that mixes a greater diversity of stimuli within the same training session. Murawska-Cialowicz et al. (2015) demonstrated that extreme conditioning programs (ECPs) (such as CrossFit) altered BDNF levels at rest, after Wingate and treadmill testing, and improved aerobic capacity of active practitioners.

In the United States, ECP training emerged in the mid-1980s, emphasizing the need to improve the physical fitness, becoming a very popular modality (Glassman, 2003; Murawska-Cialowicz et al., 2015). The ECP is a training method with a high level of effort/intensity during training sessions, in which its main goal is the improvement of the physical capacities (cardiorespiratory endurance, muscular resistance, strength, power, speed, coordination, flexibility, agility, balance, and precision). This training modality is based on Olympic weightlifting exercises (which are characterized by generating greater muscular power and the need for motor coordination), gymnastic movements (body weight or calisthenic exercises) and metabolic exercises (emphasized with cyclical movements such as running, swimming, cycling, single/double-unders and others). Also, ECP training centers is known to work with great amount of repetitions (volume), high intensity (load), high density (small periods of interval, or still absent) and small periods of stimulation (minutes), characterizing a high level of effort. Knowing that high-level effort (whether by volume or intensity) may affect the acute response of BDNF (Church et al., 2016), it is intriguing to investigate the acute response of this neurotrophin to ECPs and the influence of training parameters on BDNF changes.

According to Glassman (2010), ECP contributes to the overall development of an individual’s body and mind performance. Recently, Murawska-Cialowicz et al. (2015) demonstrated that 3 months of ECP training increased rest levels of BDNF. However, the study was performed using physically active individuals, but not previously trained in this modality. In addition, the acute parameters of BDNF were measured only using cyclic tests (Wingate and maximum progressive in cycle ergometer) and not specific ECP exercises or routine. Thus, based on their results, it is not possible to conclude that the short, voluminous and intense ECP sessions are efficient in stimulating a significant acute increase of BDNF in already trained individuals. Nevertheless, a more complete picture of the responses of this neurotrophin may indicate ECP as possible training protocol for people who are deficient in the action of this protein.

Knowing the need to understand the acute response to the specific training session of the modality, this study aimed to evaluate the acute response of BDNF in individuals trained in ECP undergoing a “workout of the day” (WOD) session. In addition, we investigated which training parameter (such as volume, load or specific exercise component) influenced the acute response of this neurotrophin.

## Materials and Methods

This study was approved by the Ethics Committee of the São Judas Tadeu University under number 37330614.7.0000.0089, in accordance with Resolution 466/12 of the National Health Council, which regulates the methodological procedures in research with human beings.

### Volunteers

Adult males who had been training ECP for at least 6 months (**Table 1**) were selected for this study (Dinoff et al., 2017). Initially, 12 individuals applied to participate in the research. After analyzing the profiles according to the inclusion and exclusion criteria, 10 were selected for the training session. This sample size is sufficient to answer our research question, as has already been demonstrated in the literature (Seifert et al., 2010; Murawska-Cialowicz et al., 2015; Church et al., 2016). Participants who were in two or more concurrent training programs and/or under the use of anabolic steroids, psychotropic drugs, antibiotics and corticosteroids, and injured were excluded.

**Table 1:** Descriptive data of the sample.

Data	Characteristics
Subjects (n°.)	10
Age (years)	31 ± 5
Height (m)	1.75 ± 0.04
Weight (kg)	83.9 ± 3.72
Training experience (months)	22 ± 9


**FIGURE 1:**
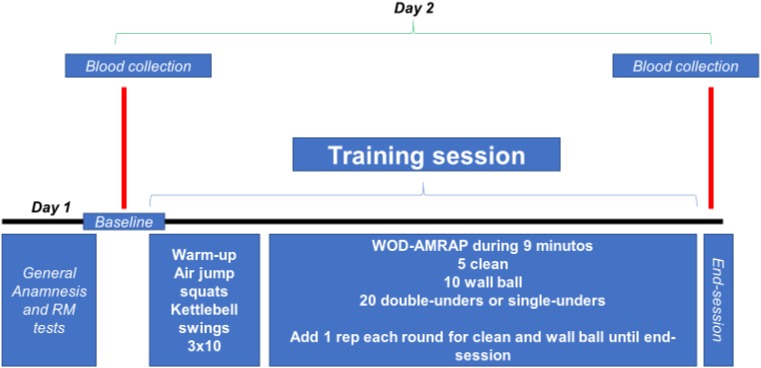
Experimental design. WOD-AMRAP: workout of the day – as many reps as possible.

All volunteers were evaluated (blood collection) at two different times: baseline (just before start the training session) and immediately after the end of the training session (**Figure 1**).

### Training Protocol

All evaluations were performed during the afternoon and each subject was submitted to the training protocol alone, in order to avoid the lack of attention to the training session and external influences (each subject was verbally stimulated only by the evaluators). All analysis was performed on 2 days, separated by 48 h, and volunteers were asked not to do any strenuous exercise for at least 72 h prior testing.

**FIGURE 2:**
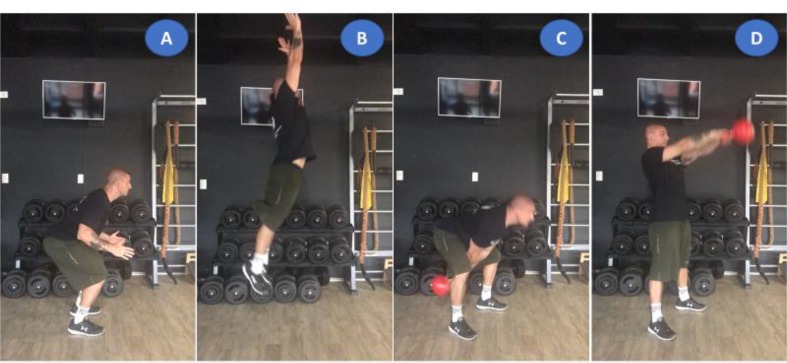
Illustration of general body warming composed of three jump air squat **(A,B)** and kettlebell swing **(C,D)** movements. The consent for both the study participation and publication of the images was both written and informed for all the participants.

**FIGURE 3:**
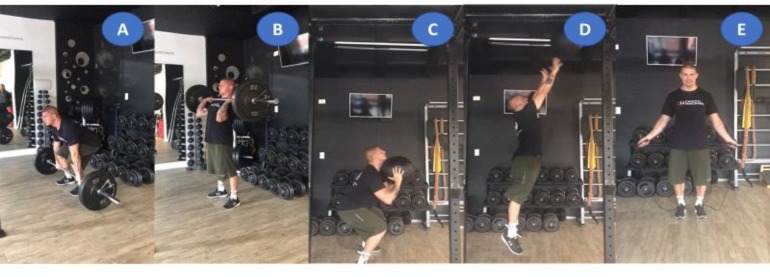
Illustration of the extreme conditioning program training session composed of clean **(A,B)**, wall ball **(C,D)**, and double-unders **(E)** exercises. Informed consent was informed, written and obtained from this volunteer for publication of their images.

On day 1, the term of free and informed consent was read together with the participants and all doubts were clarified. Also, the consent for both the study participation and publication of the images was both written and informed for all the participants (**Figures 2**, **3**). After reading and agreeing to participate in the survey, individuals respond to the International Physical Activity Questionnaire (IPAQ), the Physical Activity Readiness Questionnaire (PAR-Q) and general anamnesis. These questionnaires showed us that volunteers had the level of physical activity required in this study and that all were free of any physical limitation. Afterward, all volunteers performed a load test (RM) for clean exercise (as protocol described by Ploutz-Snyder and Giamis, 2001). Thus, the volunteers performed two sets of 5–10 repetitions, with pauses of 2–3 min. Shortly thereafter, the volunteers had up to five attempts, separated by 3–5 min, to lift the maximum load in a single movement. From this moment, we calculate the load equivalent to 80% RM for the training session. Finally, we clarify any doubts of the volunteers regarding the execution of the exercises wall ball and double or single-unders.

On day 2, thirty minutes after the baseline blood collection, all volunteers were submitted to general body warming exercises composed of 3 sets of 10 jump air squat and kettlebell swing movements (**Figure 2**). This warm-up routine was chosen by specifically preparing the requested muscle chains to follow. The training was composed of three fundamental elements in the practice of ECP (**Figure 3**): weightlifting exercise (clean), gymnastic exercise (wall ball throw) and metabolic and/or cyclic exercise (double-unders and/or single-unders).

The training session was composed of the following: workout of the day – as many repetitions as possible (method WOD-AMRAP) in 9 min: 5 repetitions of cleans, 10 repetitions of wall ball throw, and 20 repetitions of double-unders or single-unders. After each successful sets of exercises, an extra movement was added (except for double-unders and/or single-unders), making the training session more strenuous (e.g., first series 5 cleans, 10 wall ball, 20 double-unders, and/or single-unders; second series 6 cleans, 11 wall ball, 20 double-unders, and/or single-unders; and so on until the completion of time). No rest breaks were allowed during session time. The training load of the clean exercise was 80% RM (estimated in previous session) and for wall ball throw 9 kg (common load used by trained individuals for this exercise). **Figure 1** presents experimental design of this study.

To quantify the total volume (TV) and total load volume (TVL), we used the following procedures: (1) TV, we sum the total number of repetitions of the clean and wall ball exercises; and (2) TVL, the TV was multiplied by the total load of each exercise. For the calculation of TV of the double-unders exercise, the total number of repetitions performed over time of WOD-AMRAP was added. Finally, we added the volume reps of all three exercises as total training volume (TTV).

### Blood Collection

Blood samples were collected baseline (before warm-up exercise) and immediately after the training session. During the collection, the volunteers remained seated and right arm resting on a table. Approximately 10 ml of venous blood was collected in a vacuum-disposed plastic tube with no additive. After collection, the tubes were left for 30 min at room temperature and subsequently held in a styrofoam box with ice.

After the blood collection, the samples were processed in the Laboratory of Biochemistry of the São Judas Tadeu University, being centrifuged at 5,000 rpm for 15 min. After centrifugation, only the supernatant, consisting of 500 μl Eppendorf conical tube serum, was separated with identification of the individuals at each time point.

All these procedures were carried out in a clean and sterilized workbench, using the appropriate protection precautions. The samples were then stored in a freezer at -80°C.

After obtaining the biological material from all individuals, the blood samples were sent to the Laboratory of Pharmacology and Toxicology of the Faculty of Veterinary Medicine and Animal Science of the University of São Paulo, in which the serum concentrations of BDNF were measured.

Serum BDNF concentrations were measured by enzyme-linked immunosorbent assay (ELISA) technique. The technique relies on the use of antigens or antibodies labeled with an enzyme, so that the resulting conjugates have both immunological and enzymatic activity. It has one of the components (antigen or antibody) fixed on an adsorbent support, the antigen conjugate complex is immobilized, and the reaction can be easily revealed by the addition of a specific substrate that can act with the enzyme producing a color visible to the naked eye or quantifiable through the use of colorimetric and spectrophotometric techniques. The DuoSet^®^ Human BDNF kit (R&D Systems, Minneapolis, MN, United States) was used. The kit was prepared according to the manufacturer’s standards and instructions, and final results were expressed in pg/ml.

### Statistical Analysis

All results were presented as mean ± standard deviation. To test the normality of the data we used the Shapiro–Wilk test. To compare pre vs post-moments, Student’s *t*-test was performed. One-way ANOVA was used to compare the TV and TVL outcomes (*post hoc* Tukey) between exercises. For the correlation tests between TV, TVL, and the BDNF delta values, Pearson’s correlation test was used. The Cohen’s *d* effect size calculation (ES = difference between pre- and post-intervention divided by pre-intervention SD) was used to evaluate the magnitude of BDNF changes. ES values were determined from very small (0.01–0.19), small (0.20–0.49), moderate (0.50–0.79), large (0.80–1.19), and very large (1.20<). For the calculation of the data we used SPSS software version 21.0 and the level of significance was *p* ≤ 0.05.

## Results

**FIGURE 4:**
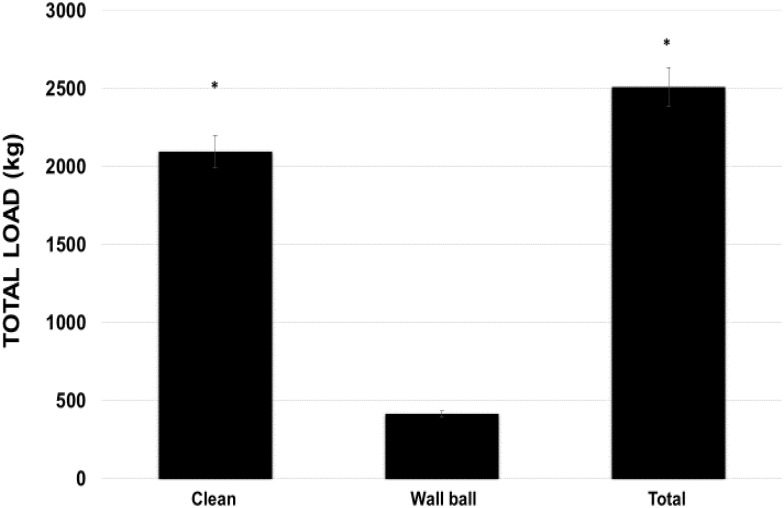
Total load volume used during the WOD (workout of the day) for the clean and wall ball exercises, and the total load of the training session. ^∗^*p* < 0.01 vs wall ball exercise. ANOVA (*F*) = 32.564, *p* < 0.05.

The TVL of the clean exercise was five times greater than wall ball exercise (*p* < 0.05; 2096.1 ± 387.4 kg vs 415.8 ± 81.03 kg), which influenced little in the total load (*p* < 0.05, 2511.9 ± 358.52 kg) used in WOD-AMRAP (**Figure 4**).

**FIGURE 5:**
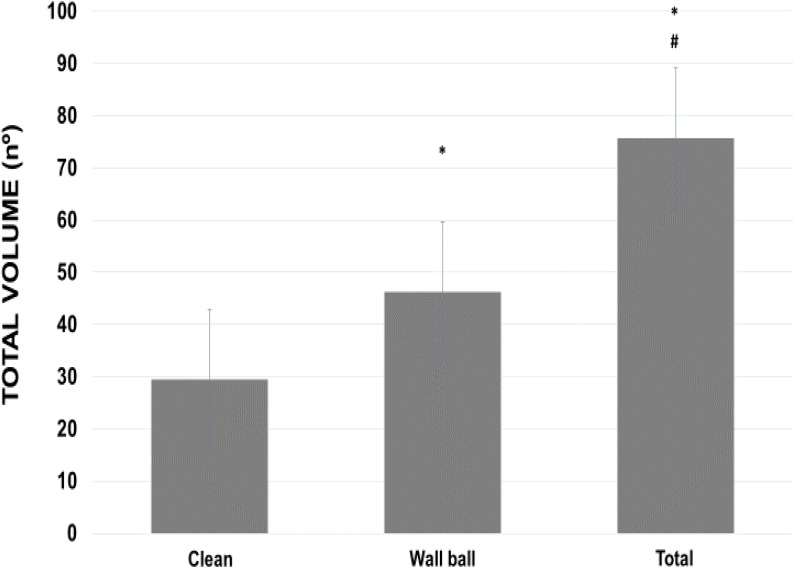
Total volume (series × repetitions) used during WOD-AMRAP (workout of the day, method as many reps as possible) for clean and wall ball exercises, and the total load of the training session. ^∗^*p* < 0.01 vs clean exercise; ^#^*p* < 0.01 vs clean and wall ball exercises. ANOVA (*F*) = 66.854, *p* < 0.01.

For the TV, practitioners averaged 1.7 times more repetitions in the wall ball compared to clean exercise (46.2 ± 9 vs 29.5 ± 3.8 repetitions). Total volume (adding both exercise repetitions) (75.7 ± 12.2 repetitions) was statistically higher (*p* < 0.01) than each exercise analyzed alone (**Figure 5**). The volunteers averaged 75.7 ± 12.6 double-unders repetitions, bringing the TTV to 151.4 ± 23.7 repetitions.

**FIGURE 6:**
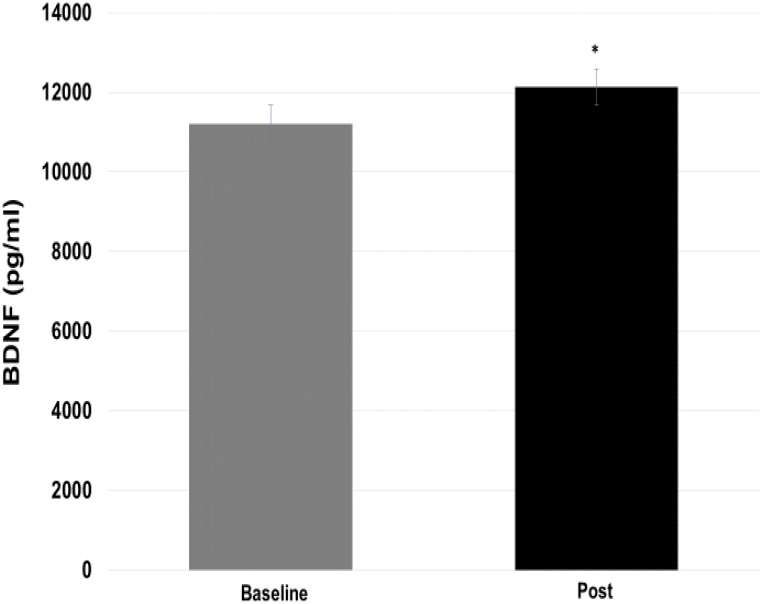
Comparison of brain-derived neurotrophic factor (BDNF) values at baseline and post-training moments. ^∗^*p* = 0.05 vs baseline.

Regarding the BDNF values (**Figure 6**), there was significant difference (*p* = 0.05) between the pre- vs post-moments (11209.85 ± 1270.4 vs 12132.96 ± 1441.93 pg/ml). The effect size for this change was shown to be moderate (ES = 0.73).

**FIGURE 7:**
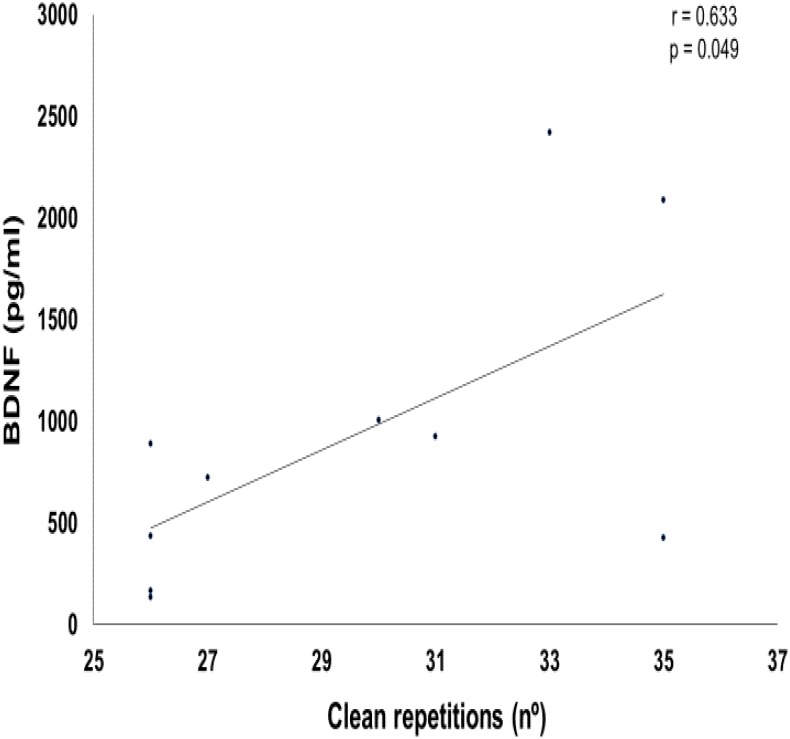
Graphic of Pearson correlation between brain-derived neurotrophic factor (BDNF) and clean exercise repetitions.

**Table 2** shows the correlation values between the training parameters (total volume and total load) and BDNF delta value changes. **Figure 7** represents a positive correlation between total volume of clean exercise and delta BDNF values (*p* = 0.049). For other comparisons, there was no significant correlation.

**Table 2:** Correlation between total volume (series × replicates) and total load (total volume × weight) of training parameters with values of delta brain-derived neurotrophic factor (BDNF) changes.

	BDNF (delta)
Total clean load (kg)	0.184
Total clean reps (n°.)	0.633^∗^
Total wall ball load (kg)	0.259
Total wall ball reps (n°.)	0.259
Total reps double unders (n°.)	0.588
Total load (kg)	0.257
Total reps clean + wall ball (n°.)	0.387
Total reps clean + wall ball + double unders (n°.)	0.512


*Asterisk “^∗^” indicates significant correlation *p* < 0.05.*

## Discussion

Here, we demonstrate two new facts: (1) as other types of exercise, a single session of ECP is significantly potent to stimulate acute increase of plasma BDNF levels, and (2) the number of repetitions of the clean exercise was positively correlated with this physiological effect.

Our results suggest that the combination of multiple types of exercise, high effort, high intensity, and high training volume in a relatively short time (9 min) can be a decisive factor in the dynamics of post-exercise changes in BDNF. Here, we confirmed results already found by other research groups, whose purpose was to investigate the effects of more common types of exercises on alterations of this neurotrophin (Seifert et al., 2010; Szuhany et al., 2015; Park and Kwak, 2017; Liu and Nusslock, 2018).

Generally, literature studies give more attention to the influence of aerobic exercise on BDNF secretion compared to predominantly anaerobic or strength exercise. Zoladz et al. (2008) observed an increase in acute levels of BDNF after a single aerobic exercise session. This phenomenon has being found in several different populations (Mackay et al., 2017; Hsueh et al., 2018; Morais et al., 2018). However, the results of our study do not seem to be influenced by the double-unders exercise (aerobic type exercise), as presented through the correlation tests. Therefore, the influence of multi-articular strength exercises on the BDNF response was fundamental. In isolation, the results are controversial as to the efficiency of strength training in increasing the plasma levels of this neurotrophin (Rojas Vega et al., 2006; Goekint et al., 2010; Yarrow et al., 2010). Possibly, the circuit training characteristic used here, was fundamental to our result. Associating the specific characteristics of the three exercises included in our study, with the high volume-intensity methodology, we demonstrated a potent effect of ECP training on BDNF levels. In general, the literature should better explore various types of program assembly in order to give practical possibilities of the results already demonstrated.

Pearson’s correlation positively pointed to the influence of the number of repetitions performed in the clean exercise on the BDNF delta. Thus, the type of exercise, the percentage of the maximum load used and the total volume of exercise training involving large muscle mass, may influence the acute responses of this neurotrophin. In accordance with our results, Lee and Soya (2017) demonstrated that acute loaded wheel running increased hippocampal BDNF activity in rats. In addition, Jeon and Ha (2017) demonstrated that moderate to high effort levels of aerobic training seems to have a positive effect on levels of BDNF at rest and on cognitive function. For the other side, Church et al. (2016) indicated that BDNF levels are increased after an acute session of resistance exercise, regardless of training differences protocols. These data confirms the need for more studies regarding the influence of different training variables on the BDNF acute responses.

Most of the exercises used here, involved moderate to high external load and could be classified as a predominantly resisted activity. Still, individuals should perform as many repetitions as possible within a predetermined time. Thus, the levels of fatigue achieved were very close to the maximum for each individual. Marston et al. (2017) used a method of training to-fatigue based resistance exercise protocol and quoted that this activity provides the necessary stimulus to increase peripheral serum BDNF. Perhaps, strenuous exercise or higher total volume may also have some influence on the outcomes measured here. Cho et al. (2014) demonstrated that although there was no difference in serum BDNF levels between aerobic and combined type exercises, higher stimulus may have a possibility of positive changes in increase of serum BDNF level in mid-aged women. Cabral-Santos et al. (2016) indicated that even different volume training would not change acute BDNF values if aerobic exercise intensity are maintained. Being so, Forti et al. (2015) showed that only a very high volume at a sufficiently high external load was able to increase circulating BDNF. These data corroborates our findings that fast and powerful movements with high intensity (80% maximum load) and higher number of repetitions can be enough to stimulate the increase in BDNF levels. Also, the amount of muscle mass used might also have an impact in the neurotrophin levels. Walsh et al. (2016) demonstrated a transient increase in serum BDNF following a single session of strength exercise in older adults after lower body resistance training. Therefore, training to voluntary fatigue might be necessary to obtain significant BDNF levels increase.

The exercises used in ECP involve the whole body, unlike those exercised in a conventional gym. Here, we include different types of exercises, such as power (clean), strength resistance (wall ball throwing) and aerobic (double-unders) exercises. This variety of exercise stimuli can activate different physiological mechanisms. According to Cassilhas et al. (2012), both exercise types (aerobic and strength) present distinct signaling pathways. Aerobic type exercise presents an increased level of IGF-1, BDNF, TrkB, and β-CaMKII (calcium/calmodulin-dependent kinase II) in the hippocampus, whereas the strength type of training showed an induction of peripheral and hippocampal IGF-1 with concomitant activation of receptor for IGF-1 (IGF-1R) and Akt in the hippocampus. These cellular pathways stimulated synapsin 1 and synaptophysin expression in both groups. These data confirmed that aerobic and strength exercise can stimulated different molecular mechanisms with similar results on learning and spatial memory. These data suggest that individuals undergoing a session, or training, using methods with various types of exercise could benefit in both signaling pathways.

Our training strategy can be effective in improving memory and learning, as seen in the prescription of more traditional exercises. In terms of applicability, this methodology can be classified as time-efficient. Thus, high-level stress sessions can be done in high training density, making it interesting as a non-medicament treatment in several degenerative dysfunctions. Tharmaratnam et al. (2018) demonstrated that 12 weeks of moderate-to-high intensity training is able to improve working memory by increasing brain oxygenation, nutrient delivery and BDNF mRNA expression in adolescents. However, higher training intensity demonstrates larger and more significant results on working memory and increase of BDNF compared to lower intensities (Jeon and Ha, 2017). Etnier et al. (2016) showed that long-term memory differed as a function of exercise intensity with better results being presented after maximal intensity levels, although BDNF increase after all exercise intensities. Pietrelli et al. (2018) presented data showing that exercise improves memory and learning thru the system serotonin-BDNF, protecting the brain against deleterious effects. These data demonstrate that both acute and chronic changes are associated with improved memory, learning and cognition, and are corroborated by other studies in several other conditions (Hopkins and Bucci, 2010; Hopkins et al., 2011; Håkansson et al., 2017). However, the large amount of data present in the literature was investigated using continuous aerobic exercise, thus requiring more investigations confirming the same effect in consideration of other types of methodologies and exercises.

Mechanisms that altered resting BDNF levels in bloodstream can be different. During exercise with high intensity or volume (as used here), lactate plasma levels are frequently increased due to the high mobilization of glycolytic metabolism. Schiffer et al. (2011) demonstrated a significant increase in BDNF levels while administering sodium lactate adult individuals. The authors quoted that lactate might participate in the regulation of BDNF production levels. However, according to Marston et al. (2017), the presence of lactate does not appear to stimulate the BDNF response during resistance exercise. Clearly, there is the need of further studies on this subject.

Further, exhaustive training sessions may stimulate increased expression and protein production of oxidative stress. Sakr et al. (2015) observed a significant augment in the levels of cortical malondialdehyde, a reduction in antioxidant activity (decreased glutathione, superoxide dismutase, and catalase) and a significant increase in BDNF expression in rats subjected to chronic effort under hypoxic conditions. These data presents evidence that oxidative stress could be an important factor stimulating BDNF expression. For the first time, Freitas et al. (2018) demonstrated a significant effect of HIIT on reducing oxidative stress, lipoperoxidation and inflammatory markers, as well enhancing antioxidant defenses and BDNF content.

In ECP sessions, adrenergic levels rise, modulating the energy response, and may stimulate feelings of well-being in the post-session. Chen and Russo-Neustadt (2007) demonstrated that norepinephrine and serotonin are responsible for inducing BDNF expression. Norepinephrine induces phosphorylation of cAMP-response element binding protein (CREB) and stimulates expression of both BDNF and nerve growth factor (NGF) (Counts and Mufson, 2010). Also, norepinephrine activates phosphatidylinositol-3 kinase (PI-3K)/Akt and mitogen-activated protein kinase (MAPK) and NO/cGMP pathways, in hippocampal neurons (Chen and Russo-Neustadt, 2013). BDNF activates the Trk receptor and participate in neuron plasticity, growth, development and survival of neurons.

Finally, the realization of ECP sessions requires high energy demand. It is fundamental to understand the action of BDNF in central and peripheral tissues. Wrann et al. (2013) link endurance exercise and the important metabolic mediators, PGC-1α and FNDC5, with BDNF expression in the brain. FNDC5, a muscle protein cleaved and secreted as irisin, is also elevated by endurance exercise in the hippocampus of rodents. Further, neuronal *Fndc5* gene expression is regulated by PGC-1α, and PGC-1α knockout mice show reduced *Fndc5* expression in the brain. Importantly, peripheral delivery of FNDC5 to the liver via adenoviral vectors, resulting in elevated blood irisin, induces expression of *Bdnf* and other neuroprotective genes in the hippocampus (Wrann et al., 2013). The findings from this work indicate that endurance exercise increase the activity of the ERRα/PGC-1α proteins, which increases levels of FNDC5 in skeletal muscle and hippocampus (Xu, 2013). Murawska-Cialowicz et al. (2015) demonstrated that 3 months of ECP training was able to increase resting BDNF levels without any alterations in irisin plasmatic concentration. This data may show that irisin may act as a paracrine regulator of BDNF.

Based on the results found here, we can conclude that a single ECP session, through the practice of the WOD-AMRAP method, is capable of increasing the acute concentrations of plasma BDNF. Secondly, we can also conclude that the total volume of repetitions of the clean Olympic exercise, when performed with high intensity (80% RM), correlates positively with the increase of BDNF found.

## Author Contributions

EP is the lead author of this manuscript, contributing all of its steps including volunteer training. WKN assisted in the steps of project preparation, statistics, data analysis, and final data preparation. AC collected the data and blood, and analyzed the BDNF. MG, LG, and CZ assisted in the assembly of the training protocol, data collection during training of the volunteers, and in the final version of the manuscript. EG guided the entire process and was present at all stages.

## Conflict of Interest Statement

The authors declare that the research was conducted in the absence of any commercial or financial relationships that could be construed as a potential conflict of interest.
