# Corrigendum: Significant Acute Response of Brain-Derived Neurotrophic Factor Following a Session of Extreme Conditioning Program Is Correlated With Volume of Specific Exercise Training in Trained Men

**DOI:** 10.3389/fphys.2019.01492

**Published:** 2019-12-10

**Authors:** Emy S. Pereira, Walter Krause Neto, Atilio S. Calefi, Mariana Georgetti, Larissa Guerreiro, Cesar A. S. Zocoler, Eliane F. Gama

**Affiliations:** ^1^Laboratory of Morphoquantitative Studies and Immunohistochemistry, Department of Physical Education, São Judas Tadeu University, São Paulo, Brazil; ^2^Laboratory of Body Perception and Movement, Department of Physical Education, São Judas Tadeu University, São Paulo, Brazil; ^3^Department of Pathology, Faculty of Veterinary Medicine and Animal Science, University of São Paulo, São Paulo, Brazil; ^4^Laboratory of Human Movement, Department of Physical Education, São Judas Tadeu University, São Paulo, Brazil

**Keywords:** high-intensity interval training, effort, strength training, aerobics, cross training, neurotrophin

In the original article, we neglected to include the funder “FAPESP, 2018/13456-6” to Eliane Florencio Gama. The **Funding** statement outlined below has now been added to the original article.

“We appreciate the support received from FAPESP, which provided funds for the publication of this article (Case No. 2018/13456-6, São Paulo State Research Support Foundation - FAPESP).”

The authors apologize for this error and state that this does not change the scientific conclusions of the article in any way. The original article has been updated.

